# 

**Published:** 1875-06

**Authors:** 


### *American Dental Convention.*

The twenty-first Annual Meeting of the American Dental Convention will be held at Long Branch, N. J., commencing on the 2nd Tuesday in August. There is no permanent membership in this organization and no initation fee. Dues are collected only from those attending the meeting. All dentists are cordially invited to be present. Essays will be read, and interesting and instructive clinics given by prominent members of the profession.

AMBLER TEES, *Rec. Secretary.*

---

### *Misisissippi State Dental Association.*

Pursuant to a call for the purpose of organizing a Dental Association, the meeting took place at the office of Miles & Askew, Vicksburg, Miss., April 21st.

Dr. J. D. Miles having been elected chairman *protem.*, and Dr. J. B. Askew Secretary, the meeting was called to order.

A copy of the constitution and by-laws for the organization having been submitted by Dr. A. H. Hilzheim, of Jackson, was unanimously adopted.

The constitution and by-laws having been signed by the members present, the meeting proceeded to the election of members, after which the following officers were elected: Drs. J. D. Miles, President; A. H. Hilzheim, 1st Vice-President; O. B. Hilzheim, 2nd Vice-President; A. Riser, Secretary; J. B. Askew, Treasurer; and an executive committee consisting of Drs. Miles, Askew and A. M. Hilzheim.

Drs. J. B. Askew, B. F. Eager, and C. M. Adams were appointed a committee of arrangements.

On motion of Dr. A. Riser, the convention adjourned to meet at Cooper's Well on the third Wednesday of August, 1875.

### *The District Dental Society.*

The District Dental Society of the seventh judicial district of the State of New York, will hold its Seventh Annual Meeting at Rochester, N. Y., Tuesday and Wednesday, June 1st and 2d, 1875.

The sessions will commence at 2 o'clock of the first day and continue until the close of the second.

Members of the profession from other Societies, States and the Dominion, are cordially invited to be present and take an active part in the discussions.

Peculiar cases, Incidents of Office Practice, Appliances and Methods of Operating, will be given particular attention.

Dentists who reside in the Seventh Judicial District, and who are not Members of this Society, may make application for Membership, on or before the first day of the meeting, to the Secretary of the Society, or to the Chairman of the Board of Censors.

Officers.—H. S. Miller, President; B. R. McGregor, Vice President; J. Edward Line, Secretary; F. E. Howard, Treasurer.

---

### *California State Dental Association.*

The Sixth Annual Session of the *California State Dental Association* will be held in San Francisco, June 8th, at 10 A. M., and will continue four days.

The sessions heretofore have been productive both of pleasure and profit; and we hope the approaching one will be characterized by as much unity of purpose and harmony of action as any of the preceding.

While visiting various portions of this coast during the last year, I have been frequently asked the object of this Association.

Its objects I will briefly state: To elevate the character and dignity of the profession; to establish uniformity in practice upon scientific principles; to develop that desire for mutual cultivation, literary research, and scientific investigation, so much needed on the part of individual members of the profession; to establish and sustain, through these liberalizing influences, the professional character of dentists; promoting among them mutual improvement, social instruction, and good feeling, these sessions affording frequent opportunities for an exchange of views in theory and practice; doing away with petty jealousies among neighboring practitioners; and promoting in their place agreeable social relations, benefiting alike patient and operator.

Much good has already been accomplished by this Association; and we do hope that none of its members will "become weary of well-doing."

Its chief object being mutual improvement, social intercourse, and the elevation of the profession, therefore, every member should feel himself called upon to make some sacrifices in order to attend its annual meetings. It is due to himself, to his patients, and the community in which he resides; and in view of these obligations, we sincerely hope every one will make an effort to be present.

During the session there will be a great variety of subjects introduced and discussed of great vital importance to all members of the profession. These discussions are demonstrated by clinical operations in filling teeth, etc., it being a fact admitted by all that there is more practical informaation obtained by clinical operations than by any other means, for it is truly astonishing what rapid progress some of our profession have made by having the opportunity of seeing operations by the most experienced class of operators.

Members of the profession, you cannot afford to remain away from these sessions of the Association. If you do, you cannot give the full benefit of your progressive profession to your patients.

Hoping that you will honor us with your presence at our meeting, I remain,

H. H. Pierson,

*President California State Dental Association.*

### TO READERS AND CORRESPONDENTS.

Communications intended for publication, and exchange journals and papers, should be addressed to the EDITOR OF THE AMERICAN JOURNAL OF DENTAL SCIENCE, 259 N. Eutaw St., Baltimore, Md. All letters relating to the business of the journal, or containing cash remittances, to be directed to Messrs. SNOWDEN & COWMAN. Articles for publication should be received by the editor at least three weeks previous to the first day of the month on which the number in which it is designed for them to appear, is issued.

### CONTENTS:

### ORIGINAL COMMUNICATIONS.

ARTICLE I.—Notes on Celluloid. By James B. Hodgkin, D. D S.......... 49
ARTICLE II.—Prosecution for Dental Fees.......... 52
ARTICLE III.—Obituary.—Dr. M. McCarty. By S. J. Cobb, D. D. S.......... 62
ARTICLE IV.—What may our Patients Reasonably Expect shall be Accomplished by Operations upon the Teeth. By Geo. H. Cushing, D. D. S.......... 63
ARTICLE V.—A Dentist in the Newspapers.......... 68
ARTICLE VI.—A Biographical Sketch of the Life of Dr. T. B. Hamlin. By S. J. Cobb, D. D. S.......... 72

### SELECTED ARTICLES.

### MONTHLY SUMMARY.

Treatment of Cold.......... 93
Nelaton's Method of Resuscitation.......... 94
Antidote to Chloroform.......... 94
Solvents for Rubber.......... 95
Singular Death from inhaling Ether.......... 95
Death after Intra-venous Injection of Chloral.......... 96
Intra-venous Injection of Chloral for Producing Anæsthesia.......... 96

☞The American Journal of Dental Science will contain fifty pages of reading matter in each number, making a volume of not less than

*Six Hundred* pages

EXCLUSIVE OF ADVERTISEMENTS.

THE

### AMERICAN JOURNAL

OF

### DENTAL SCIENCE.

F. J. S. GORGAS, M. D., D. D. S., Editor.

The *Journal* is issued on the first of each month and contains not less than *fifty* pages of reading matter in each number, exclusive of advertisements, making a volume of *six hundred* pages per annum.

In each number will be found Original Communications, Correspondence, Selected Articles, Monthly Summary, Bibliographical Notices and Editorial Matter, and every effort will be exerted to render the *Journal* worthy of the patronage of the Dental profession.

A generous co-operation is respectfully solicited from the members of the profession; and as the *Journal* has now a printing office of its own, which materially lessens the cost of its publication, it will be issued at the reduced subscription price of

**$2.50 Per Annum in Advance.**

Communications are respectfully solicited on all subjects pertaining to the practice of dentistry, as well as reports of cases occurring in dental practice.

### RATES OF ADVERTISING.

| | 1 MO. | 2 MO. | 3 MO. | 6 MO. | 12 MO. |
|---|---|---|---|---|---|
| 1 PAGE. | $15 00 | $22 50 | $30 00 | $50 00 | $100 00 |
| ½ " | 10 00 | 15 00 | 20 00 | 30 00 | 50 00 |
| ¼ " | 7 00 | 11 00 | 15 00 | 20 00 | 30 00 |
| ⅛ " | 5 00 | 8 00 | 11 00 | 15 00 | 20 00 |

All original communications designed for publication, works for review, new instruments for notice, exchanges, &c., should be directed to the editor, 259 N. Eutaw St., Baltimore, Md.

All advertisements and subscriptions should be addressed to

SNOWDEN & COWMAN,
*Publishers of the Am. Journal of Dental Science.*
86 W. FAYETTE ST. BET. CHARLES & LIBERTY STS.,
BALTIMORE, MD.

